# Supplementary material for: Iterative improvement in the automatic modular design of robot swarms
Source: PeerJ Comput Sci. 2020 Dec 7;6:e322. doi: 10.7717/peerj-cs.322 (PMC7924708; doi:10.7717/peerj-cs.322)
Supplement: Supplemental Information 3 [file peerj-cs-06-322-s003.zip › argos3/doc/api/standalone/a00617_source.html]

ARGoS: plugins/simulator/physics\_engines/dynamics2d/dynamics2d\_velocity\_control.cpp Source File


- Main Page
- Related Pages
- Namespaces
- Classes
- Files

- File List
- File Members

# plugins/simulator/physics\_engines/dynamics2d/dynamics2d\_velocity\_control.cpp

Go to the documentation of this file.

```
00001 
00007 #include "dynamics2d_velocity_control.h"
00008 #include <argos3/plugins/simulator/physics_engines/dynamics2d/dynamics2d_engine.h>
00009 
00010 namespace argos {
00011 
00012    /****************************************/
00013    /****************************************/
00014 
00015    CDynamics2DVelocityControl::CDynamics2DVelocityControl(CDynamics2DEngine& c_engine,
00016                                                   Real f_max_force,
00017                                                   Real f_max_torque) :
00018       m_cDyn2DEngine(c_engine),
00019       m_ptControlBody(NULL),
00020       m_ptControlledBody(NULL),
00021       m_ptLinearConstraint(NULL),
00022       m_ptAngularConstraint(NULL),
00023       m_fMaxForce(f_max_force),
00024       m_fMaxTorque(f_max_torque) {
00025       m_ptControlBody = cpBodyNew(INFINITY, INFINITY);
00026    }
00027 
00028    /****************************************/
00029    /****************************************/
00030 
00031    CDynamics2DVelocityControl::~CDynamics2DVelocityControl() {
00032       cpBodyFree(m_ptControlBody);
00033    }
00034 
00035    /****************************************/
00036    /****************************************/
00037 
00038    void CDynamics2DVelocityControl::AttachTo(cpBody* pt_body) {
00039       /* If we are already controlling a body, detach from it first */
00040       if(m_ptControlledBody != NULL) {
00041          Detach();
00042       }
00043       /* Set the wanted body as the new controlled one */
00044       m_ptControlledBody = pt_body;
00045       /* Add linear constraint */
00046       m_ptLinearConstraint =
00047          cpSpaceAddConstraint(m_cDyn2DEngine.GetPhysicsSpace(),
00048                               cpPivotJointNew2(m_ptControlledBody,
00049                                                m_ptControlBody,
00050                                                cpvzero,
00051                                                cpvzero));
00052       m_ptLinearConstraint->maxBias = 0.0f; /* disable joint correction */
00053       m_ptLinearConstraint->maxForce = m_fMaxForce; /* limit the dragging force */
00054       /* Add angular constraint */
00055       m_ptAngularConstraint =
00056          cpSpaceAddConstraint(m_cDyn2DEngine.GetPhysicsSpace(),
00057                               cpGearJointNew(m_ptControlledBody,
00058                                              m_ptControlBody,
00059                                              0.0f,
00060                                              1.0f));
00061       m_ptAngularConstraint->maxBias = 0.0f; /* disable joint correction */
00062       m_ptAngularConstraint->maxForce = m_fMaxTorque; /* limit the torque */
00063    }
00064 
00065    /****************************************/
00066    /****************************************/
00067 
00068    void CDynamics2DVelocityControl::Detach() {
00069       if(m_ptControlledBody != NULL) {
00070          /* Remove constraints */
00071          cpSpaceRemoveConstraint(m_cDyn2DEngine.GetPhysicsSpace(), m_ptLinearConstraint);
00072          cpSpaceRemoveConstraint(m_cDyn2DEngine.GetPhysicsSpace(), m_ptAngularConstraint);
00073          /* Free memory up */
00074          cpConstraintFree(m_ptLinearConstraint);
00075          cpConstraintFree(m_ptAngularConstraint);
00076          /* Erase pointer to controlled body */
00077          m_ptControlledBody = NULL;
00078       }
00079    }
00080 
00081    /****************************************/
00082    /****************************************/
00083 
00084    void CDynamics2DVelocityControl::Reset() {
00085       m_ptControlBody->v.x = 0;
00086       m_ptControlBody->v.y = 0;
00087       m_ptControlBody->w = 0;
00088    }
00089 
00090    /****************************************/
00091    /****************************************/
00092 
00093    CVector2 CDynamics2DVelocityControl::GetLinearVelocity() const {
00094       return CVector2(m_ptControlledBody->v.x,
00095                       m_ptControlledBody->v.y);
00096    }
00097 
00098    /****************************************/
00099    /****************************************/
00100 
00101    void CDynamics2DVelocityControl::SetLinearVelocity(const CVector2& c_velocity) {
00102       m_ptControlBody->v.x = c_velocity.GetX();
00103       m_ptControlBody->v.y = c_velocity.GetY();
00104    }
00105 
00106    /****************************************/
00107    /****************************************/
00108 
00109    Real CDynamics2DVelocityControl::GetAngularVelocity() const {
00110       return m_ptControlBody->w;
00111    }
00112 
00113    /****************************************/
00114    /****************************************/
00115 
00116    void CDynamics2DVelocityControl::SetAngularVelocity(Real f_velocity) {
00117       m_ptControlBody->w = f_velocity;
00118    }
00119 
00120    /****************************************/
00121    /****************************************/
00122 
00123 }
```

---

Generated on 10 Jul 2018 for ARGoS by 
 1.6.1
